# STAT3 pathway as a molecular target for resveratrol in breast cancer treatment

**DOI:** 10.1186/s12935-021-02179-1

**Published:** 2021-09-06

**Authors:** Zeynab Kohandel, Tahereh Farkhondeh, Michael Aschner, Ali Mohammad Pourbagher-Shahri, Saeed Samarghandian

**Affiliations:** 1 Department of Biology, Faculty of Sciences, University of Tehran, Tehran, Iran; 2 Cardiovascular Diseases Research Center, Birjand University of Medical Sciences, Birjand, Iran; 3 Faculty of Pharmacy, Birjand University of Medical Sciences, Birjand, Iran; 4 Department of Molecular Pharmacology, Albert Einstein College of Medicine, Bronx, New York, USA; 5 Noncommunicable Diseases Research Center, Neyshabur University of Medical Sciences, Neyshabur, Iran

**Keywords:** STAT3, Breast cancer, Malignancy

## Abstract

Signal transducer and activator of transcription 3 (STAT3) induces breast cancer malignancy. Recent clinical and preclinical studies have demonstrated an association between overexpressed and activated STAT3 and breast cancer progression, proliferation, metastasis, and chemoresistance. Resveratrol (RES), a naturally occurring phytoalexin, has demonstrated anti-cancer activity in several disease models. Furthermore, RES has also been shown to regulate the STAT3 signaling cascade via its anti-oxidant and anti-inflammatory effects. In the present review, we describe the STAT3 cascade signaling pathway and address the therapeutic targeting of STAT3 by RES as a tool to mitigate breast cancer.

## Introduction

Resveratrol (RES), also known as trans-3,4′,5-trihydroxystilbene, was first extracted from the white hellebore roots in 1939 by Michio Takaoka [1]. It is ubiquitous in various plants, including grape skin and by inference in wine, berries, peanuts, p*olygonum cuspidatum*, and *Rheum officinale Baill*, which are traditional Chinese medicines [2, 3]. RES has been used to mitigate inflammation, reduce oxidative stress, suppress growth, and increase apoptosis [4]. These properties have led to its use as a therapeutic agent in multiple disorders such as cancer, cardiovascular, inflammatory, and neurodegenerative diseases [5]. RES exerts its efficacy through different molecules and pathways [6]. One of the target factors of RES is signal transducers and activators of transcription 3 (STAT3), an important transcription factor in several cancers, including breast cancer [7].

**Fig. 1:**
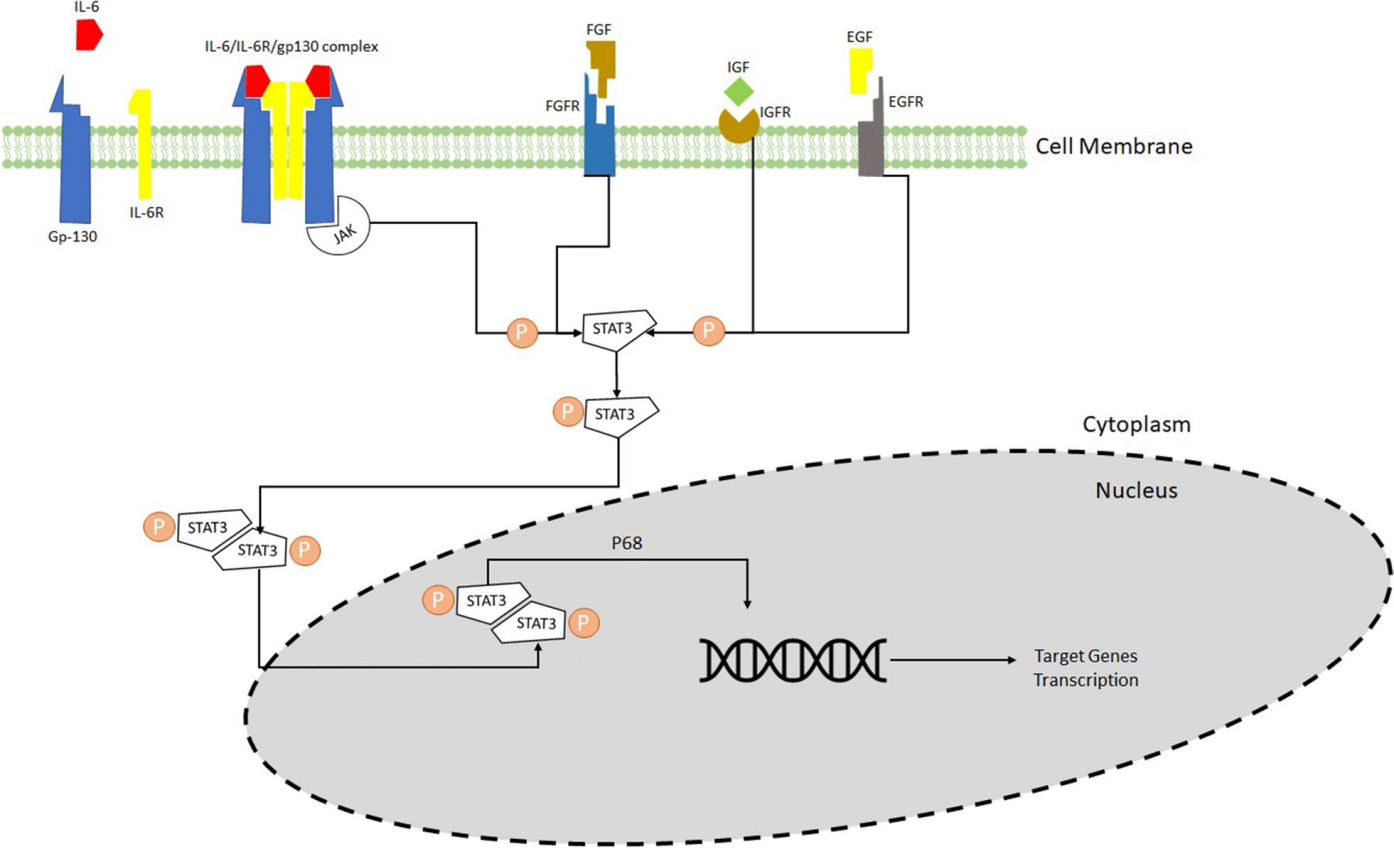
The classical STAT3 Signaling Pathway in Cancer Cells. Binding of IL-6 to its membrane-bound IL-6 receptor α (IL-6R) and IL-6 receptor β (also known as gp130) results in formation of IL-6/IL-6R/gp130 complex. The IL-6/IL-6R/gp130 complex activates the JAKs by phosphorylation which results in STAT3 phosphorylation and activation. STAT3 activation can be achieved by phosphorylation via other factors such as growth factors (i.e. FGF, IGF and EGF). These growth factors bind to their cognate membrane receptors. Upon phosphorylation, STAT3 forms a homodimer which translocates into the nucleus to bind to the promotor region of target genes and activates target gene transcription

Among cancer-related deaths globally, breast cancer is the second most common in women [8]. Only few treatment approaches are available for it, given chemoresistance to most treatment modalities [9]. Various transcription factors (TFs) are directly associated with breast cancer progression and development [10, 11]. The STAT family comprises seven TFs, STAT1, STAT2, STAT3, STAT4, STAT5a, STAT5b, and STAT6, among the most critical TFs in breast cancer. These TFs are structurally similar and highly conserved [11–13]. Overall, six functional domains have been recognized, comprised of an N-terminal (NH2) domain, also known as STAT_int, DNA-binding (DBD) domain, SRC homology 2 (SH2) domain, coiled-coil (CDC) domain, transactivation (TAD) domain, and linker domain [14]. The involvement of STAT3 in the development, metastasis, multidrug resistance, and proliferation of cancer has been subject to extensive research [15, 16]. Classical STAT3 signaling cascade has been shown in several investigations [7, 17, 18]. Figure 1 provides an overview of STAT3 signaling cascades. As noted, several cytokines, such as interleukin 6 (IL-6) and interleukin 10 (IL-10), and growth factors comprising epidermal growth factor (EGF), fibroblast growth factor (FGF) and insulin-like growth factor (IGF), initiate the activation of STAT3 [19, 20]. Binding of these elements to their relevant receptors, leads to the activation of Janus kinases (JAKs) [21]. Cognate receptor’s cytoplasmic tail is phosphorylated by JAKs, followed by binding the SH2 domain of STAT3 to phosphorylated tyrosine residues. The phosphorylation of STAT3 enables the translocation of signals from the cytoplasm to the nucleus by forming homodimers. Once translocated, pSTAT3 binds to the target genes’ promoter site, by shaping a complex with several coactivators, such as p68, and leading to their transcription [22]. In the present review, we summarize the role of RES, via its effects on STAT3, in the development of breast cancer, focusing on the most recent and pertinent studies.

**Fig. 2:**
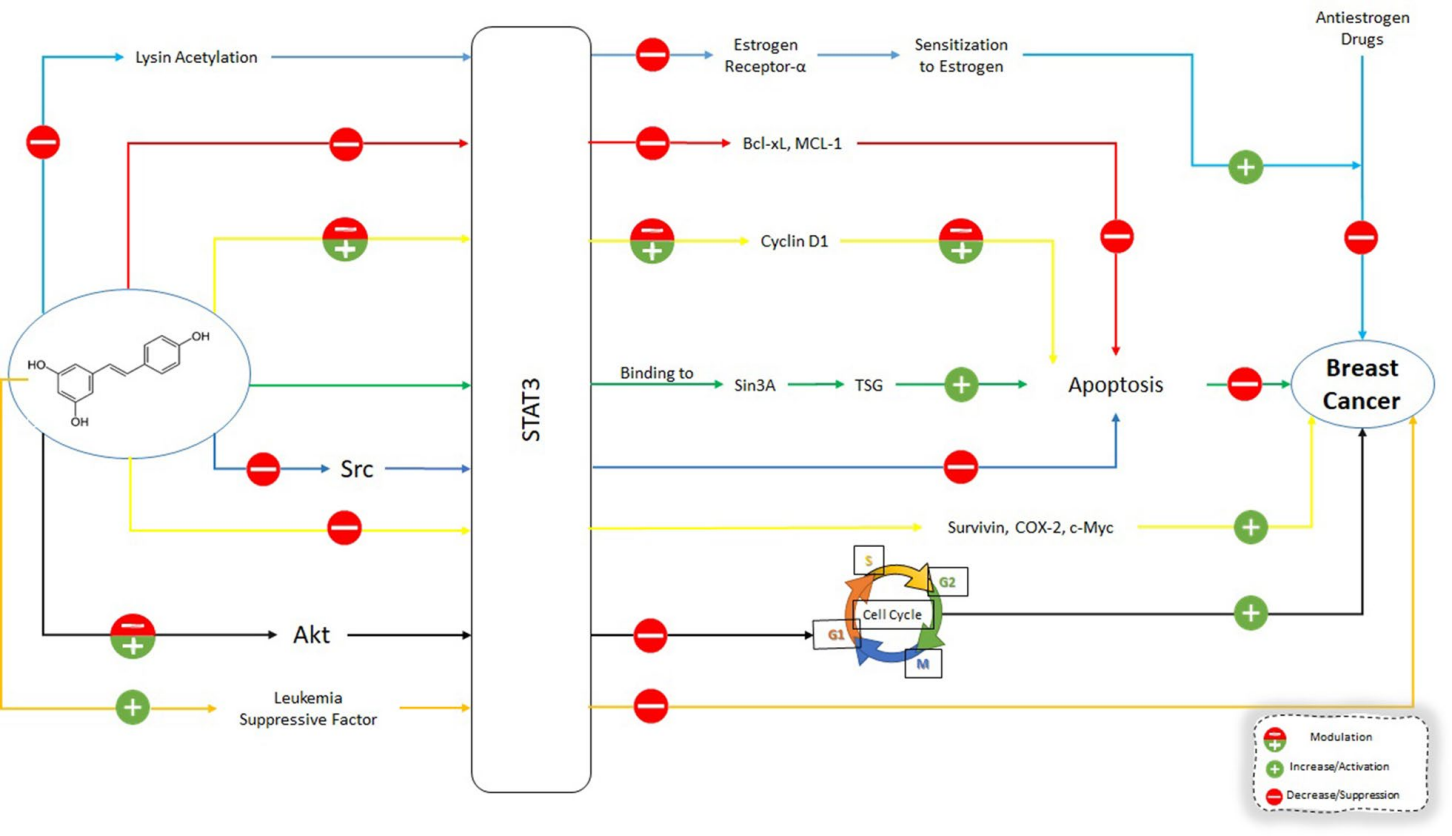
Effects of Resveratrol on STAT3 Signaling Pathway in Breast Cancer Cells. Resveratrol can directly affect the STAT3 and its downstream molecular targets in breast cancer cells. Also, resveratrol can affect upstream regulators of STAT3 which results in changes in downstream molecular targets affecting the growth, progression and metastasis of breast cancer cells. Bcl-xL: B-cell lymphoma-extra large; MCL-1: Myeloid cell leukemia-1; TSG: tumor suppressor genes; COX-2: Cyclooxygenase-2; Akt: Protein kinase B (PKB) aka Akt

The role of STAT3 in breast cancer progression, apoptosis, metastasis, proliferation, and chemoresistance will be discussed first. Once the interplay between STAT3 and breast cancer is delineated, next, the effect of RES on STAT3, and finally, the effects of RES on breast cancer through STAT3 will be addressed (Fig. 2).

### STAT3 and breast cancer progression

Oncostatin M (OSM), a cytokine and a member of the IL-6 family, can upregulate and phosphorylate IL-6 and STAT3, respectively, advancing breast cancer progression [23]. OSM can also activate STAT3 and hypoxia-inducible factor-1 alpha (HIF-1α) in estrogen receptor (E.R.)- breast cancer cells or in ER + breast cancer cells in concert with IL-6 [24]. Moreover, other factors enhance breast cancer progression, including several interleukins, such as IL-35 and IL-8. IL35 suppresses conventional T cells (T-conv). It induces of breast cancer progression by activating STAT1 and STAT3 [25]. In contrast, IL-8 and growth-regulated oncogene (GRO) chemokines induce the activation of STAT3 and inflammatory breast cancer progression [25]. Activation of STAT3 has been shown to prevent breast cancer progression by diminishing the expression of IL-17 [26].

There are several other regulators for STAT3, comprised of suppressors, such as microRNA and protein tyrosine phosphatase 2 (PTPN2), as well as activators, such as prostaglandin E2, cyclooxygenase-2 (COX2), SET and MYND (myeloid-Nervy-DEAF-1) domain-containing protein (SMYD2). In recent studies, epigenetics mediators have also been investigated and recognized as STAT3 mediators in the genesis of breast cancer. A class II histone deacetylase mediates upregulated activation of STAT3 in breast cancer, histone deacetylase 6 (HDAC6), as well as prostaglandin E2 and COX2 [27]. Activated STAT3 binds to the COX2/ prostaglandin (PGE)2 gene promoter and leads to the activation of COX-2 and PGE2 expression.

Further investigations have shown that STAT3 methylation or phosphorylation is regulated by lysine methyltransferase SMYD2, resulting in breast cancer progression [28]. Cyclin-dependent kinases 4/6 (CDK4/6) interacts with SMYD2, in turn, leading to the phosphorylation and activation of SMYD2, and resulting in SMYD2 mediating STAT3 methylation.

microRNAs (miR) have also been of great interest in the study of cancer progression and development. Nuclear enriched abundant transcript 1 (NEAT1) has been shown to cause breast cancer progression by a feedback loop with STAT3, and miR-124 has been shown to inhibit NEAT1 [29]. Hosea et al. posited that glucosamine leads to the prevention of STAT3 activation followed by a reduction in breast cancer stemness and progression [30]. Moreover, EGF-modulated STAT3 activation has been noted upon PTPN2 knockdown.

Chronic inflammation is involved in breast cancer progression, and it can be suppressed by STAT3 inhibition [31]. However, stat3 signaling cascades are also regulated by several other modulators. For example, inflammation and progression of breast cancer and the elevation of forming breast cancer stem cells are mediated through the IL-6/STAT3/ROS pathway [32]. Additionally, the FAM3 cytokine family includes an oncogenic member, TGFβ-modulated FAM3C/Interleukin-like EMT Inducer (ILEI), which can lead to the formation of breast cancer stem cells, accelerating the progression of breast cancer [8, 9]. Furthermore, STAT3 has been shown to regulate TNFRSF1A, a gene encoding one of the transmembrane receptors for TNF-α, resulting in the activation of NF-κB signaling in breast cancer [33].

It has also been shown that the progression and the proliferation of breast cancer are affected by several STAT3 co-factors. For example, progranulin (PGRN) has been shown to impart chemoresistance and worsen breast cancer prognosis [34, 35]. In agreement, progranulin antisense oligonucleotide has been shown to suppress STAT3 oncogenesis in CRC cells [36]. The cyclin-dependent kinase 5 (CDK5) regulatory subunit-related protein 3 (CDK5RAP3, also known as C53/LZAP), has been considered as a p53 co-activator [37]. It has been recently shown that CDK5RAP3 is associated with the proliferation and progression of breast cancer, ameliorating STAT3-dependent genes expression [38]. Therefore, targeting the STAT3 co-factors might represent a novel therapeutic modality in the regulation of breast cancer.

### STAT3 and metastasis

One of the critical factors in breast cancer metastasis is matrix metallopeptidases (MMPs). One of the most investigated STAT3-modulated mechanisms is via the upregulation of MMP2, MMP9, Twist, Snail, Slug, and vimentin [39–41]. It has been demonstrated that vasodilator-stimulated phosphoprotein (VASP), MMP2, and MMP9 expression in breast cancer is diminished upon repression of STAT3 phosphorylation [42]. The binding of cytokines and growth factors with their cognate receptors on the plasma membrane induces the activation of STAT3 signaling. The inhibition of receptor binding by Wwox suppresses metastasis in breast cancer [43]. Additionally, induction of JAK/STAT3 signaling cascade activation, mesoderm-specific transcript (MEST) stimulates Twist expression [44], while inhibition of the JAK/STAT3 and protein kinase B (Akt) cascade, GRAM domain-containing protein 1B (GRAMD1B) prevents breast cancer cell migration [45].

Further, it has been shown that OSM/SMAD3 induces the activation of STAT3 and modulates the expression of Snail. It also induces epithelial-mesenchymal transition (EMT) in breast cancer, instead of erstwhile binding of ligand/receptor in the plasma membrane for the activation of STAT3, alluding to a novel mechanism for the activation of STAT3 via cytoplasmic molecules and endogenous signaling [46]. Other molecules regulate STAT3-modulated breast cancer metastasis, such as proto-oncogene serine/threonine-protein kinase (PIM1), miRNA, Mucin-1-C (MUC1-C), natriuretic peptide receptor A (NPRA), and RhoU. Han et al. have demonstrated that modulating the exogenous signaling cascade, known as Krüppel-like factor 11 (KLF-11), induces the activation of STAT3 by binding to its transmembrane receptor KLF-11R, while miR-30d regulates breast cancer cell migration and invasion [47]. Furthermore, the JAK/STAT3 cascade in breast cancer bone metastasis is modulated by IL-11 [48]. The IL-6/STAT3 signaling cascade modulates PIM1, a proto-oncogene that induces cell invasion and upregulates the expression of EMT in breast cancer [49]. An oncogenic protein, MUC1-C, has also been shown to activate STAT3 and stimulate Twist transactivation, leading to the induction of EMT [50]. Additionally, a natriuretic peptide receptor, NPRA, increases STAT3 and MMP9 expression, resulting in the induction of breast cancer cells migration and invasion [51]. STAT3 also stimulates the expression of high Ras Homolog Family Member U (RhoU) by collaborating with Specificity Protein 1 (SP1), leading to breast cancer cell migration [52].

Moreover, upon STAT3 posttranscriptional changes, several enzymes are effective in breast cancer metastasis. Dai et al. have demonstrated that the induction of STAT3 phosphorylation and the elevation of MMP2 and MMP9 expression in breast cancer cells are stimulated by Rac-specific Rho GTPase-activating protein (Rho GAP), also known as ARHGAP24 [53]. Zhao et al. have shown that the upregulation of p-STAT3, p-AKT, MMP9, and E2F1 expression by a histone acetyltransferase, called GCN5, leading to breast cancer migration and invasion [54].

Hypoxia is a state of stress that is widely investigated in cancer. Hypoxia can stimulate STAT3 activation, causing the promotion of breast cancer stemness and metastasis [55]. These findings afford new approaches for research on STAT3 therapy targets in breast cancer. In addition, it has been recently shown that estrogen-associated receptor alpha may induce triple-negative breast cancer metastasis as a STAT3 target gene [56].

### STAT3, proliferation, and apoptosis

Apoptosis has a central role in the pathogenesis of several cancers; therefore, it is necessary to identify the underlying mechanism of apoptosis in cancer [57]. Active STAT3 in tumor cells contributes to the preservation of tumor cells by inhibiting apoptosis [58]. Considerable evidence corroborates the presence of active STAT3 in greater than 95% of cancers, leading to diminished apoptosis [59].

Stat3 protein activation by Src has been shown to be required for cell transformation and promotes cell proliferation.

There is a significant association between high anti-apoptotic proteins such as BCL-2 and Bcl-xL and cancer [60]. IL- 6 mediated Stat3 signaling to induce Bcl-xL gene expression [61]. In addition, over-expression of Bcl-xL protects cells from the anti-apoptotic effect of Stat3 signaling.

Recently, a clinical study indicated that constitutive STAT3 activation caused the expression of Mcl-1 and Bcl-xL, resulted in cancer cell progression and survival [62]. Evidence indicated that Stat3 regulates several genes related to the promotion of cell cycle progression and inhibition of the apoptotic pathway, leading to tumorigenesis [63]. In breast cancer cells, activation of STAT3 via the IL-6/JAK2 cascade causes suppression of Bax/Bcl-2-associated caspase-dependent apoptosis [64]. STAT3 has been shown to upregulate cyclin D-1, c-myc, and bcl-2, leading to the prevention of breast cancer cells apoptosis, suggesting an association between STAT3 in cell cycle and survival [65]. Down-regulation or up-regulation of several mi-RNAs can promote the activation of STAT3 signaling pathways in breast cancer cells. In this regards, it was found that down-regulation of DPF3 [64], miR-125a and let-7e [66], miR-124 [67], miR-9 [68] can activate STAT3 signaling pathway. In contrast, up-regulation of several miRNAs, including miR-93-5p and miR-25-3p, have been shown to induce the proliferation of breast cancer by activating STAT3 [69].

### STAT3 and chemoresistance

Studies in triple-negative breast cancer cells have shown that Src/STAT3 signaling cascade is associated with multidrug resistance (MDR) [70]. Castellaro et al. have demonstrated that by activating the NF-κB/STAT3/ERK cascade, an association between breast cancer cells and macrophages stimulates tamoxifen and ICI 182,780 resistance [71]. There are several identified STAT3-modulated chemoresistance downstream targets, such as carnitine palmitoyltransferase 1B (CPT1B), fatty acid beta-oxidation (FAO), mitogen-activated protein kinase (MAPK)/AKT, HIF-1, and octamer-binding transcription factor-4 (Oct-4). Wang et al. have shown increased CPT1B and FAO levels, secondary to activation of the JAK2/STAT3 signaling cascade, leading to breast cancer chemoresistance [72]. It has also been noted that activation of the JAK-STAT3/MAPKs/AKT cascade, IL-22 stimulates the migration and paclitaxel resistance in breast cancer [73]. Furthermore, via the STAT3/hypoxia-inducible factor 1 (HIF-1) cascade target, miR-124 causes the reversion of breast cancer cells doxorubicin (DOX) resistance [74]. Cheng et al. investigated the formation of a signal circuit via Oct-4 and c-myc concerning increased Adriamycin resistance in breast cancer [75]. On the other hand, IL-24 generation in breast cancer cells via STAT3 and NF-B-activation, confers radiation resistance by Oct-4 [76]. Moreover, phosphorylation of STAT3 has been shown to regulate survivin, in turn conferring resistance to paclitaxel, which is extensively used in breast cancer treatment [77].

Several upstream modulators of STAT3-regulated chemoresistance have been recognized. Upon STAT3 activation, the COOH-terminal proline-rich area of 78-kDa glucose-modulated protein (GRP78), has been shown to play a vital role in the progression of tamoxifen-resistant breast cancer cells [78]. Moreover, induction of STAT3 activation caused by leukemia inhibitory factor receptor (LIFR) leads to resistance to Trastuzumab-emtansine (T-DM1) in breast cancer [79]. Feng et al. also showed that miR-4532 promotes resistance to Adriamycin in breast cancer by inhibiting hypermethylated in cancer-1 (HIC-1) and IL-6/STAT3 [80].

On the other hand, several other small molecules mediate chemoresistance regulated by STAT3. For example, Chen et al. have found that via JAK/STAT3 cascade, a combination of Piperlongumine with DOX stimulates apoptosis and suppresses breast cancer cells’ DOX resistance [81]. Furthermore, utilizing the combination of STAT3 suppressor with a poly ADP-ribose polymerase (PARP) suppressor, IL6/STAT3 activity could be targeted, resulting in the successful treatment of palbociclib resistance in breast cancer cells [82].

### RES phenotypically influences breast cancer

As we previously mentioned, RES, a natural polyphenolic phytoalexin, exerts anti-inflammatory and anti-oxidative activity. RES administration is associated with anti-breast cancer efficacy. RES has a leading role against breast cancer cell growth at all stages, including initiation, promotion, and progression [83]. In addition, its anti-oxidant and anti-inflammatory activities enable RES to inhibit the development of breast cancer by inducing apoptosis and cell cycle arrest. RES’s estrogenic activity also contributes to its protective effects against breast cancer cell growth modulating estrogen receptor activity [84].

RES was effective against different molecular subtypes of breast cancer, including luminal A [ER+, PR+/-, Her-2-, low Ki 67]; luminal B [ER+, PR±, Her-2+, high Ki 67], E.R. -, Her-2 + and basal [E.R. -, P.R. -, Her-2-, EGFR + and cytokeratin 5/6+, high Ki 67] [85].

### RES effects on the upstream and the downstream of STAT3 pathways

In vivo investigations have indicated that RES suppresses tumor growth and stimulates apoptosis by inhibiting STAT3 [86]. In this context, Kotha et al. have shown that RES suppresses the activation of Src tyrosine kinase. In turn, it causes the inhibition of constitutive activation of Stat3 protein in tumorigenesis cells [40]. Further, they reported that malignant cells with constitutively active STAT3, treated with RES, showed irreparable cell cycle arrest at the G0-G1 phase or the S phase of several cancer cell lines, including human breast (MDA-MB-231) cell line, as well as apoptosis, leading to loss of viability [40]. On the other hand, they observed that RES administered to cells that did not include abnormal STAT3 activity, led to reversible growth arrest and a loss of viability. Additionally, RES administration to malignant cells with constitutively induced active STAT3 caused inhibition of STAT3-modulated cyclin D1, and Bcl-xL, and Mcl-1 genes, suggesting that the anti-cancer activity of RES is mediated by blocking STAT3-modulated dysregulation of growth and survival pathways [40].

Yu et al. reported the potential effect of RES in STAT3 signaling cascade in two cancer cell lines [87]. The expression and phosphorylation of STAT3 were observed in regular cultured cell lines, with RES reducing both parameters. Furthermore, the expression of downstream genes of STAT3, including survivin, cyclin D1, Cox-2, and c-Myc, was prevented. concomitantly, Bcl-2 was elevated secondary to RES administration [87]. Moreover, the production and secretion of a STAT3 activator, leukemia suppressive factor, was activated in RES-treated cells. Overall, these findings suggest STAT3 signaling cascade might be a significant target for RES [87] Li et al. have shown that RES can suppress cell proliferation through G1 phase cell cycle arrest and induce cell apoptosis in cancer cells, while not affecting normal cells, indicating that the AKT/STAT3 pathway may represent a novel target for RES [88].

It was also found that RES inhibited proliferation, migration, and invasion of human breast cancer cells exposed to cancer-associated fibroblast-conditioned media (CAF-CM) by suppressing the CAF-CM-induced expression of Cyclin D1, c-Myc, MMP-2 and MMP-9, Sox2 expression and also the activation of Akt and STAT3. Additionally, RES decreased the expression of self-renewal signaling molecules in stem-like breast cancer cells [89].

The endothelial to mesenchymal transition (EndMT) is responsible for cancer metastasis via modulating the complexity of the tumor microenvironment (TME). RES, an inhibitor of STAT3 acetylation, reduced the expression of Ac-STAT3, p-STAT3, and EndMT markers in human umbilical vein endothelial cells (HUVECs) exposed to 27-hydroxycholesterol (27HC). In addition, inhibition of STAT3 signaling by RES prevents Cross-talk between 27HC-induced EndMT in the TME [90].

RES blocks Stat3 activation by inhibiting the Src tyrosine kinase activity in human breast cancer cells, leading to dysregulation of growth and survival pathways [91].

In tumor cells, an increase in the lysine acetylation of the STAT3 induces cell growth. It was indicated that genetically modifying STAT3 at Lys685 decreased tumor growth due to demethylation and reactivation of several tumor-suppressor genes. Treatment with RES decreased acetylated STAT3 in triple-negative breast cancer cells resulted in demethylation and activation of the estrogen receptor-α gene, sensitizing the breast cancer cells to antiestrogens [92]. RES reduced STAT3 acetylation and inhibited tumor suppressor genes (TSG) expression, leading to apoptosis in breast cancer cells. STAT3 bound the Sin3A transcriptional repressor. This complex bound the promoter region of silenced TSG following Res administration into the breast cancer cells [93]. 6-methyl-2-propylimino-6, 7-dihydro-5 H-benzo [1, 3]-oxathiol- 4-one (LYR71), a derivative of trimeric resveratrol, was also effective against breast cancer growth via inhibiting the STAT3-mediated MMP-9 expression [94].

## Conclusions

This novel review highlights STAT3 as a critical transcriptional activator in breast cancer, which can mediate breast cancer progression, metastasis, chemoresistance, apoptosis, and proliferation. Several of its upstream regulators and downstream targets are inhibitable by naturally occurring substrates, such as RES. Specifically, we highlighted studies that show the propensity of RES to suppress breast cancer cells proliferation and induce apoptosis via the STAT3 signaling pathway. Taken together, modulation of STAT3 levels should be further explored as a potential novel pharmaceutical modality for the treatment of breast cancer as well as other cancer types.

## Data Availability

The authors confirm that the data supporting the findings of this study are available within the article.
